# *glpx* Gene in *Mycobacterium tuberculosis* Is Required for In Vitro Gluconeogenic Growth and In Vivo Survival

**DOI:** 10.1371/journal.pone.0138436

**Published:** 2015-09-23

**Authors:** Hiten J. Gutka, Yuehong Wang, Scott G. Franzblau, Farahnaz Movahedzadeh

**Affiliations:** 1 Institute for Tuberculosis Research, College of Pharmacy, University of Illinois at Chicago, Chicago, Illinois, United States of America; 2 Department of Medicinal Chemistry and Pharmacognosy, College of Pharmacy University of Illinois at Chicago, Chicago, Illinois, United States of America; University of Delhi, INDIA

## Abstract

Several enzymes involved in central carbon metabolism and gluconeogenesisplay a critical role in survival and pathogenesis of *Mycobacterium tuberculosis (Mtb)*. The only known functional fructose 1,6-bisphosphatase (FBPase) in *Mtb* is encoded by the *glpX* gene and belongs to the Class II sub-family of FBPase. We describe herein the generation of a *ΔglpX* strain using homologous recombination. Although the growth profile of *ΔglpX* is comparable to that of wild type *Mtb* when grown on the standard enrichment media, its growth is dysgonic with individual gluconeogenic substrates such as oleic acid, glycerol and acetate. In mice lung CFU titers of Δ*glpX* were 2–3 log_10_ lower than the wild-type *Mtb* strain. The results indicate that *glpX* gene encodes a functional FBPase and is essential for both in vitro and in vivo growth and survival of *Mtb*. Loss of *glpX* results in significant reduction of FBPase activity but not complete abolition. These findings verify that the *glpX* encoded FBPase II in *Mtb* can be a potential target for drug discovery.

## Introduction

*Mtb* grows on a variety of substrates in vitro but mounting evidence indicates that during infection most of its energy is derived from fatty acids [1, 2]. When bacterial metabolism is fueled by fatty acids, synthesis of sugars from intermediates of the TCA cycle (particularly the glyoxylate shunt) become important for growth and persistence [3–6]. Hence, the glyoxylate shunt enzymes malate synthase and isocitrate lyase are considered potential targets for the development of new antibacterial agents [6, 7]. Phosphoenolpyruvate carboxykinase (PEPCK), the enzyme linking the TCA cycle and gluconeogenesis, catalyses the reversible decarboxylation and phosphorylation of oxaloacetate (OAA) to form phosphoenolpyruvate (PEP). The PEPCK-encoding gene *pckA* is up-regulated by acetate or palmitate, but down-regulated by glucose. Deletion of the *pckA* gene of *Mycobacterium bovis* BCG led to a reduction in the capacity of the bacteria to infect and survive in macrophages [8]. PEPCK plays a pivotal role in the pathogenesis of *Mtb*, as it is essential for growth and survival of this pathogen during infections in mice and *Mtb* relies primarily on gluconeogenic substrates for in vivo growth and persistence [9]. Except for these recent studies, the role of gluconeogenesis in *Mtb* pathogenesis has remained largely unaddressed. Therefore, understanding the key structural and functional aspects of enzymes in the gluconeogenic pathway becomes important. Using genetic and biochemical methods, Movahedzadeh *et al*. have identified *Rv1099c* gene as a *glpX* gene that encodes a class II FBPase [10]. Fructose-1,6-bisphosphatase (FBPase, EC 3.1.3.11), a key enzyme of gluconeogenesis, catalyzes the hydrolysis of fructose-1, 6-bisphosphate to form fructose 6-phosphate and orthophosphate. This reaction is the opposite of that catalyzed by phosphofructokinase in glycolysis. Fructose 6-phosphate is the catalytic product of fructose 1,6-bisphosphate, an important precursor in various biosynthetic pathways generating important structural components of the cell wall and glycolipids in mycobacteria. Gluconeogenesis is an important metabolic pathway in several organisms, that allows the cells to synthesize glucose from noncarbohydrate precursors such as organic acids, amino acids, and glycerol. FBPases are members of the large superfamily of lithium-sensitive phosphatases, which includes both the inositol phosphatases and FBPases. These enzymes demonstrate bivalent metal-dependent and lithium-sensitive phosphatase activity [10]. Five different classes of FBPases have been identified based on their amino acid sequences (FBPases I to V) [11–14]. Eukaryotes possess only the FBPase I-type enzyme, but all five types exist in various prokaryotes. Many organisms have more than one FBPase, mostly the combination of types I and II. The type I FBPase is the most widely distributed and is the primary FBPase in *Escherichia coli*. An additional class II FBPase is encoded by the *glpX* gene in *E*. *coli*, which is part of the glycerol 3-phosphate regulon [11]. The completion of the genome sequence of *Mtb* allowed the identification of genes that were predicted to encode enzymes for most central metabolic pathways [15]; however, no FBPase was initially assigned. Results from genetic and biochemical analyses revealed that the *Rv1099c* gene of *Mtb* encodes the missing mycobacterial FBPase (II) [10]. The protein encoded by the *Mtb glpX* (*Rv1099c*) gene is Similar to other class II FBPase from *E*. *coli* (GlpX) (42% identity) and *Corynebacterium glutamicum* FBPase II (65% identity) [16]. The genome wide transposon site hybridization (TraSH) experiment [17] suggests the *glpX* gene of *Mtb* to be essential for survival in a mice infection model. Although the method is high throughput and genome wide, it does not characterize individual transposon mutants. To investigate the role of this enzyme in mycobacterial pathogenesis, we generated an unmarked *glpX* gene deletion knock out (KO) in *Mtb* and investigated its effect on in vitro and in vivo growth and survival behavior.

## Materials and Methods

### Cloning and mutagenesis of *Mtb glpX* gene

Mutagenesis was essentially carried out as previously described [18]. The coding sequence of *glpX* (*Rv1099c*) with flanking DNA, 893bp upstream and 970bp downstream of the gene, was amplified by PCR and cloned into p2NIL [18], producing pFM143 (Table 1). The primers used (GlpX_1 and GlpX2) are listed in Table 2. To create a deletion in pFM143, 943bp were deleted in the *glpX* gene by inverse PCR, using glpX_Rev1 and glpX_Rev2 primers (Table 2)., The PCR product was re-ligated producing FM147. The deletion created was confirmed by sequencing. Following insertion of a gene cassette carrying the *lacZ* and *sacB* genes from pGOAL19 into the vector’s PacI site (producing FM152); the DNA was introduced into *M*. *tuberculosis* H37Rv by electroporation. Mutagenesis was carried out as previously described [18]. To confirm the mutagenesis, colony PCR and Southern blot were performed.

**Table 1:** Strains and plasmids used for the generation of Δ*glpx* and *glpx* complement strains.

Strains	Characteristics	Source
*M*. *tuberculosis* H37Rv	wild-type laboratory strain	ATCC 27294
HG1	*M*. *tuberculosis ΔglpX*	This study
HG2	*M*. *tuberculosis ΔglpX*::pFM163	This study
*E*.*coli* DH5α		Invitrogen
p2NIL	manipulation vector	[18]
pGOAL19	delivery cassette vector	[18]
pBluescript II SK+		Stratagene
pUC-Gm-int		[19]
pFM143	p2NIL::*glpx*	This study
pFM147	pFM143::*glpXΔ*	This study
pFM152	pFM147/with PacI cassette of pGoal19	This study
pFM158	pBluescript SK+::*glpX* (445/bp upstream)	This study
pFM163	pFM158::*intgm*	This study

**Table 2:** List of primers used for the generation of Δ*glpx*.

Glpx_Rev1	AGCGCCGTGTACCCATTGCC
Glpx_Rev2	GTCACCCGGACCAGCTCCAT
tbglpX_up	GCTCTGGGTCAAGCTCAGAT
tbglpX_end	GGGCAATGGGTACACGGC

### Complementation of Δ*glpX*

A fragment containing the *glpX* gene together with 445 bp of upstream sequence was produced by PCR of *M*. *tuberculosis* genomic DNA using primers tbglpX_up and tbglpX_end (Table 2), and The PCR product was cloned into the SmaI site of pBluescript-SK+ to produce pFM158. The *Hin*dIII cassette of pUC-Gm-int, carrying the *int* and *gm* genes was cloned into the *Hin*dIII site of pFM158 to produce pFM163. The plasmid was electroporated into HG1, yielding HG2.

### Bacterial Strains, media and growth conditions

*ΔglpX*, *glpX* complement and WT *Mtb* H37Rv strains were grown using the shaker flask method in 7H9 liquid medium containing 0.2% glycerol, 10% OADC (Oleic acid, Albumin, Dextrose and Catalase), and 0.05% Tween 80. For growth with defined carbon sources, 7H9 medium was used with 0.05% Tyloxapol and a carbon substrate at 0.1% or 0.2% (wt/vol).

### In vivo growth and survival profile of Δ*glpX* strain in mice model

BALB/c mice (approximately eight weeks old) were used for evaluating the effect of *glpX* gene knockout on in vivo growth. For each strain, 36 mice were infected via aerosol delivery. At indicated time points 6 mice from each group (infected with the respective strain) were sacrificed via carbon dioxide asphyxiation and the lungs from individual mice were aseptically removed, homogenized and CFU were determined.

### Cellular FBPase activity in *Mtb* strains

Bacteria were grown to mid-log phase, disrupted in a bead beater, the cell-extracts were clarified and FBPase assays were conducted as described previously [20].

### Hydrogen peroxide sensitivity

*Mtb* cultures were grown in 7H9 medium + OADC (100ml) until late log or early stationary phase (Klett units ~ 150) and then treated with 1ml of 500mM hydrogen peroxide solution (effective peroxide concentration = 5mM). 1 ml samples were taken as controls before hydrogen peroxide treatment (referred to as 0 hr /input sample). Additional samples (1 ml) were taken at 2 hrs, 6 hrs and 12 hrs after hydrogen peroxide treatment.

### pH sensitivity

Late log or early stationary phase of 100 mL *Mtb* cultures were treated with 6ml of sterile 2 mM acetic acid solution (effective pH ≈ 4.5). 1 ml sample was taken as control before addition of acetic acid (referred to as 0 hr /input sample). Additional 1ml samples were taken at 2 days, 4 days and 6 days after pH change to ≈ 4.5 units.

### Nitrosative stress

Late log or early stationary phase of 100 mL *Mtb* cultures were treated with 10ml of sterile 2.2 mM DETANO (Diethylenetriamine/nitric oxide) solution (effective DETANO concentration = 0.2 mM). 1ml sample was taken as control before DETANO treatment (referred to as 0 Hr /input sample). Additional 1ml samples were taken at 1 day, 2 days and 3 days after DETANO treatment.

### MIC

MIC values for standard drugs were obtained using the standard Microplate-based Alamar Blue Assay (MABA) assay [21].

## Results

### Unmarked deletion mutant of *glpX* gene in *Mtb*

**Fig 1:**
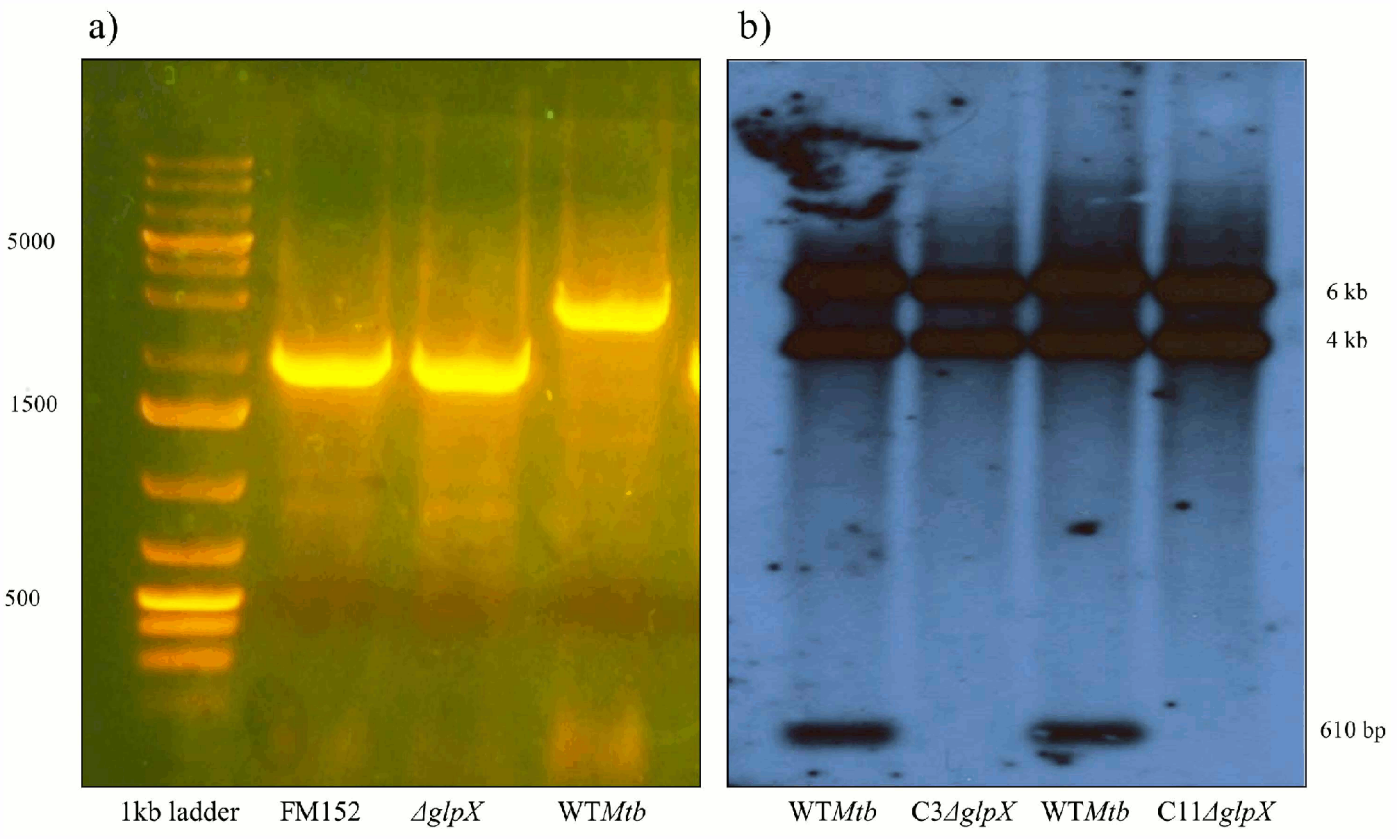
Colony PCR and Southern Blot confirming the deletion of *glpX* gene a) Colony PCR results of the potential double cross overs (DCOs): Lane 1: 1kb ladder, Lane 2: PCR amplification product of suicidal delivery vector FM152 is about 2kb, Lane 3: PCR product of one such potential DCO (Colony 11) is 2kb, Lane 4: PCR product of a WT Mtb is about 3kb, b) Southern blot: Lane 1: WT genomic DNA digest with BamH1 which gives a fragment of about 610 bp (lowermost band in lane 1 and 3), Lane 2: Genomic DNA digest of a potential DCO (Colony 3) with the 610 bp fragment missing, Lane 3: Same as Lane 1, Lane 4: Genomic DNA digest of a potential DCO (Colony 11) with the 600 bp fragment missing.

An in-frame deletion of *glpX (Rv1099c)* was constructed in *M*. *tuberculosis* H37Rv producing strain HG1. PCR and Southern blotting analysis were used to confirm the presence of the mutation (Fig 1).

### In vitro growth profile of *ΔglpX* strain (glycerol, dextrose and acetate as carbon sources)

**Fig 2:**
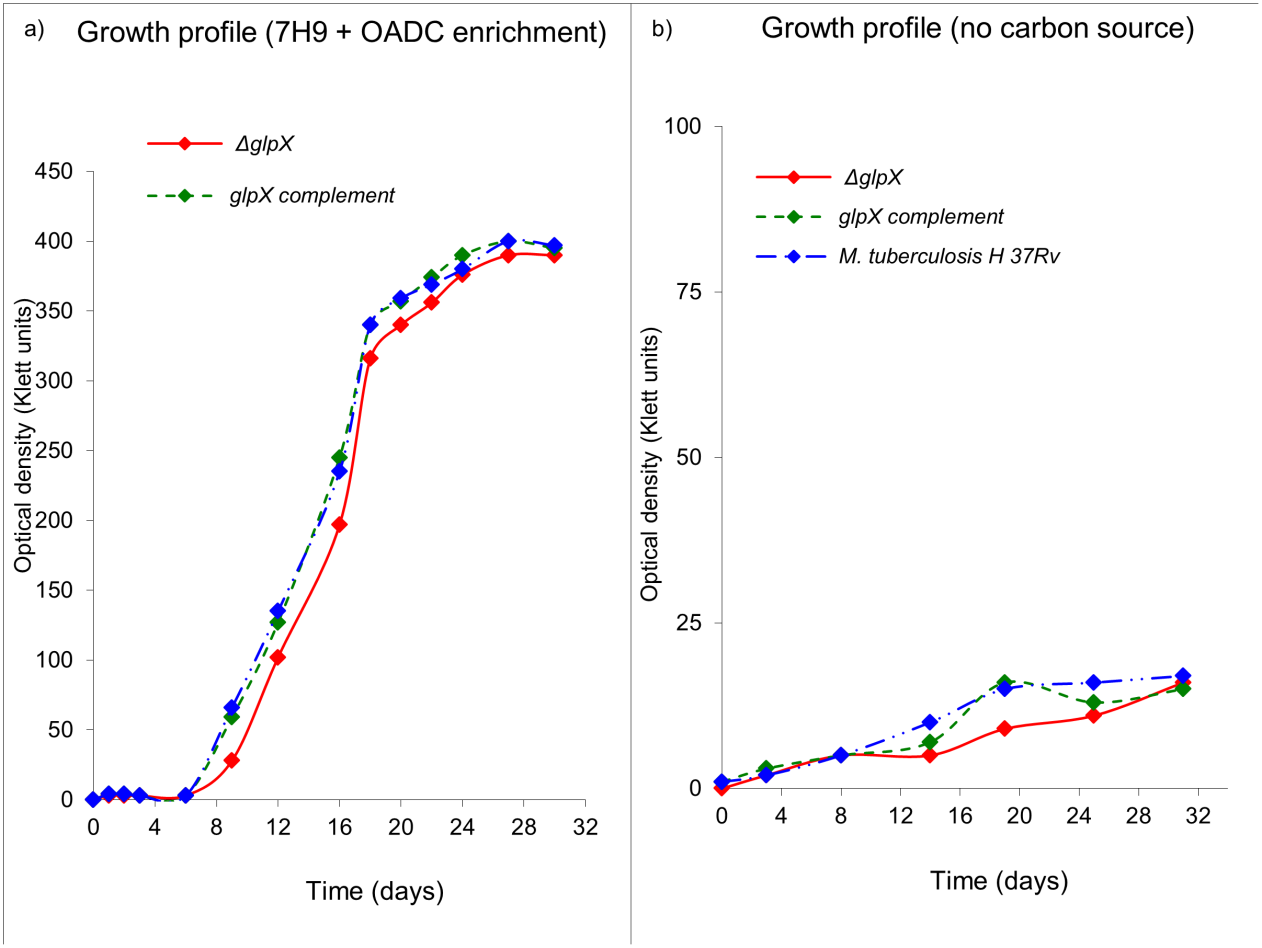
In vitro growth profile of *ΔglpX*, WT *Mtb* and *glpX* complement in a) 7H9 medium + OADC enrichment, b) 7H9 medium with no additional carbon source. Growth profiles are representative of a triplicate data set.

The growth profile of *ΔglpX* is similar to that of WT *Mtb* when grown in 7H9 medium with OADC enrichment, plus glycerol and Tween 80, a surfactant which can furnish *Mtb* with oleic acid (a fatty acid carbon source) through de-esterification [22]. Fig 2 indicates that the disruption of *glpX* does not significantly affect the growth profile of *Mtb*, in enriched media (Fig 2a). Both *ΔglpX* and WT *Mtb* failed to grow significantly in the absence of any external carbon source (glycolytic/gluconeogenic) as indicated in the growth profile (Fig 2b).

**Fig 3:**
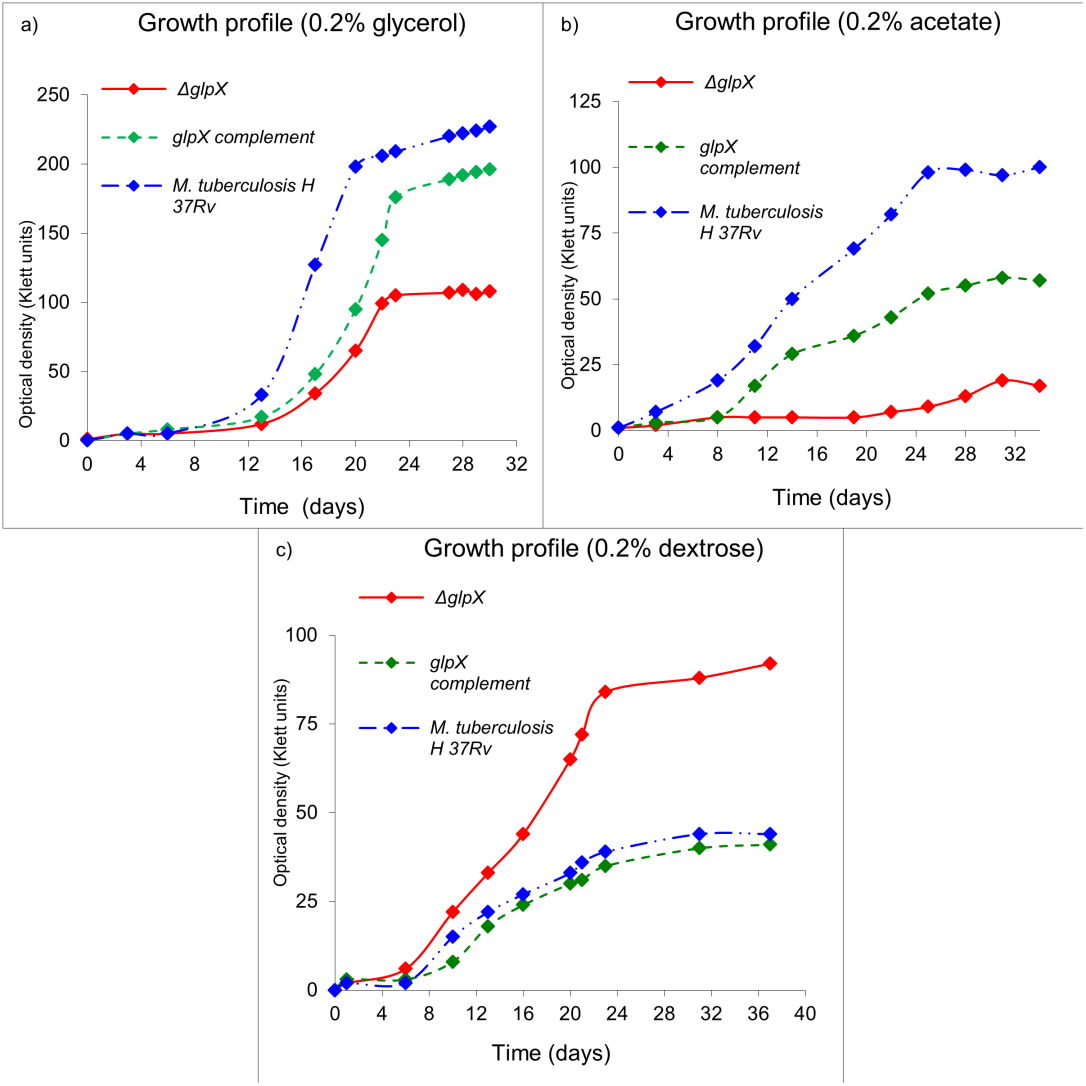
In vitro growth profile of *ΔglpX*, WT *Mtb* and *glpX* complement on defined (or individual) carbon source(s). Growth profile in 7H9 medium a) 0.2% glycerol, b) 0.2% Acetate, and c) 0.2% dextrose. Growth profiles are representative of a triplicate data set.

For studies involving growth of *ΔglpX* on defined carbon sources, defined media was used containing equal concentrations of dextrose, acetate or glycerol as single carbon sources and replacing Tween 80 with a non-hydrolysable detergent, Tyloxapol (Fig 3).

### In vitro growth profile of *ΔglpX* strain on fatty acids as a sole carbon source

**Fig 4:**
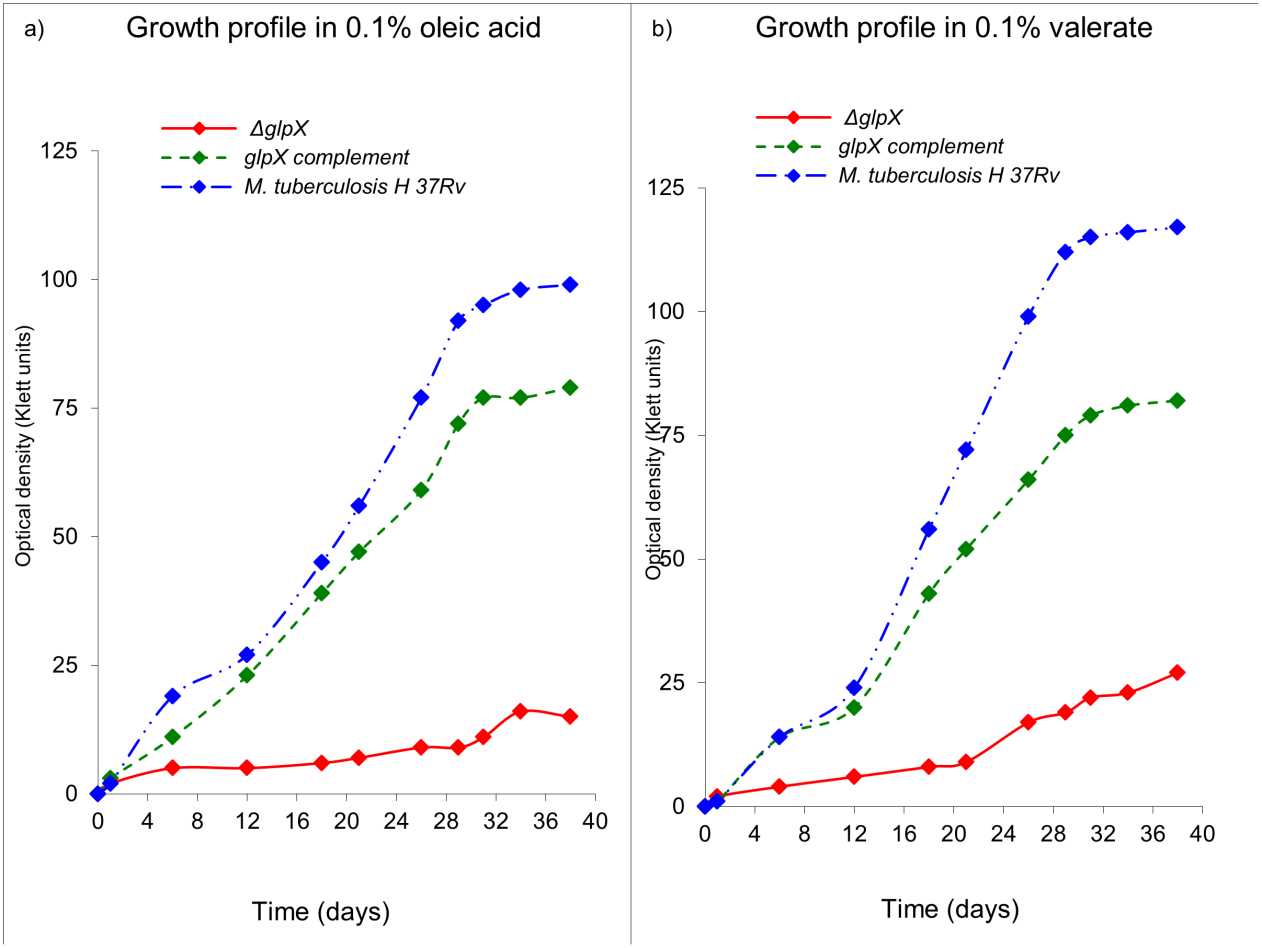
In vitro growth profile of *ΔglpX*, WT *Mtb* and *glpX* complement on gluconeogenic carbon source(s). Growth profile in 7H9 medium a) 0.1% oleic acid, and b) 0.1% valeric acid Growth profiles are representative of a triplicate data set.

As expected and similar to acetate and glycerol based growth profiles, the growth of *ΔglpX* was severely compromised (Fig 4) on oleic acid (C18) (Fig 4a) or valeric acid (C5) (Fig 4b) as a sole carbon source, further substantiating the observation with glycerol.

### In vitro growth profile of *ΔglpX* strain on combination of carbon sources

**Fig 5:**
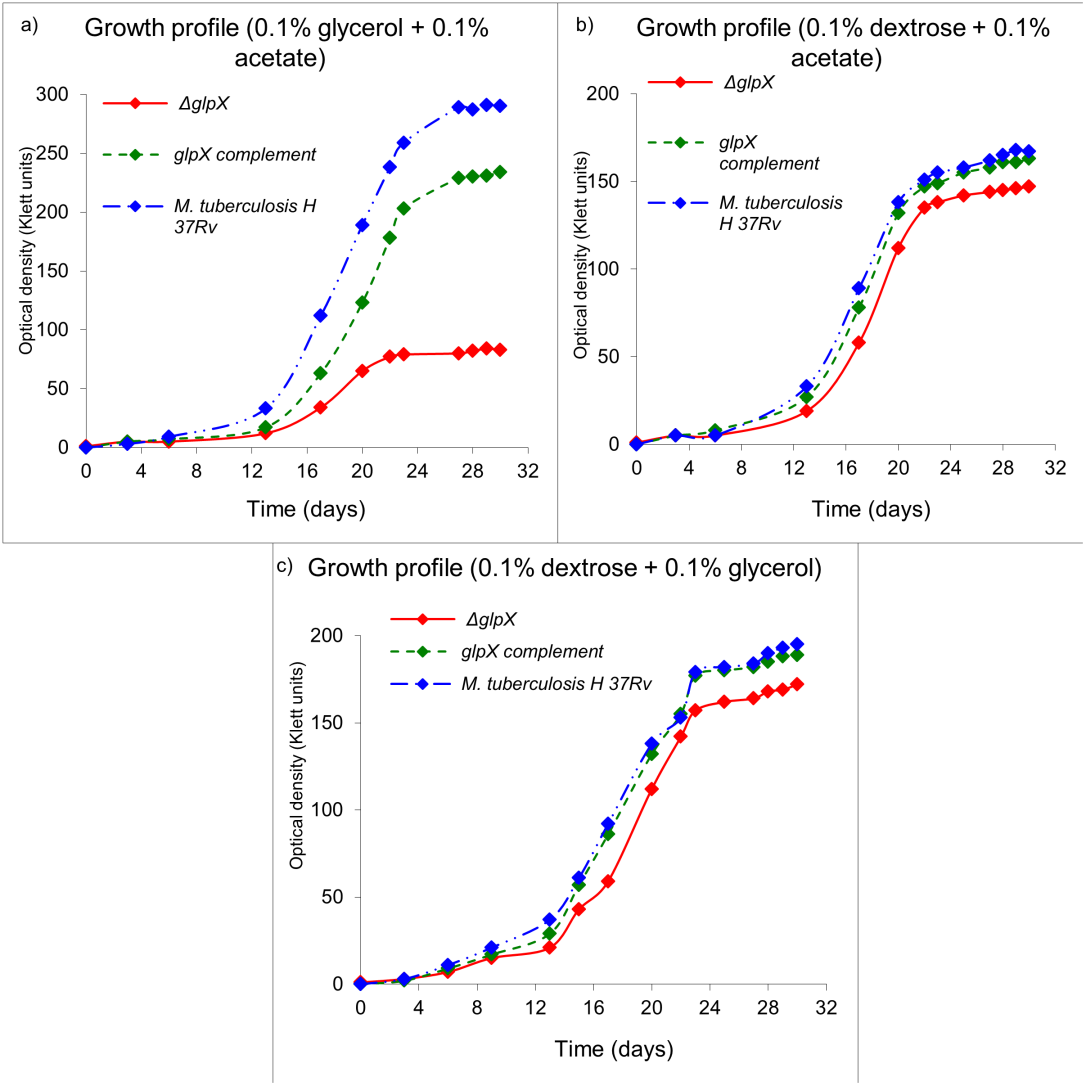
In vitro growth profile of *ΔglpX*, WT *Mtb* and *glpX* complement on combination of carbon sources. Growth profile in 7H9 medium and a) 0.1% each of glycerol and acetate, b) 0.1% each of dextrose and acetate, and c) 0.1% each of dextrose and glycerol. Growth profiles are representative of a triplicate data set.

Growth was subsequently monitored using combinations of two carbon sources including acetate, dextrose and glycerol respectively (Fig 5).

### *glpX* is essential for growth in acute phase and survival during chronic phase of *Mtb* infection in mice

**Fig 6:**
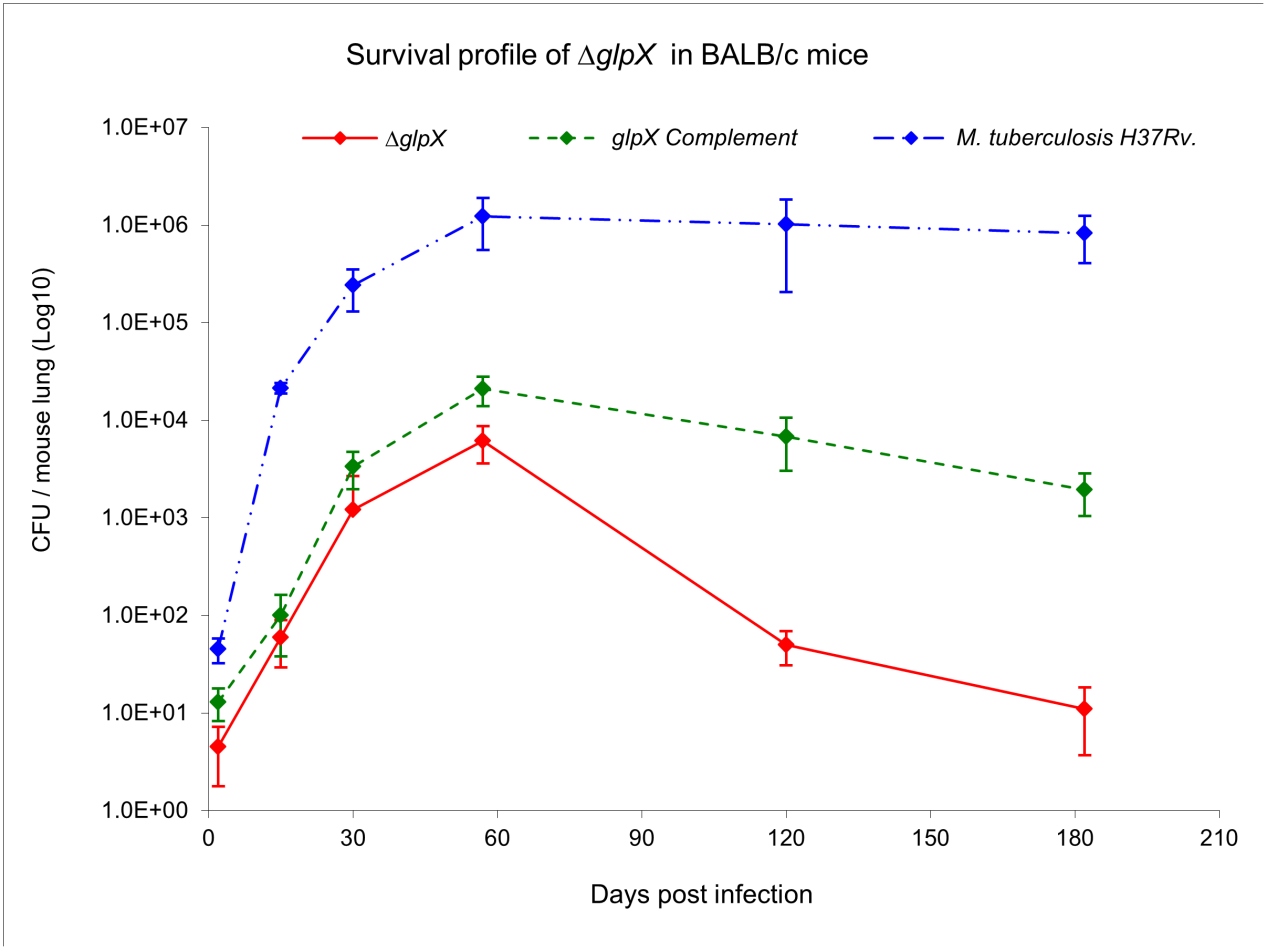
*glpX* is essential for growth in acute phase and survival during the chronic phase of *Mtb* infection in mice. Invivo growth and survival plot for *ΔglpX* compared to WT *Mtb* and the *glpX* complement. Data represents the mean±s.d. of six mice per time point.

**Fig 7:**
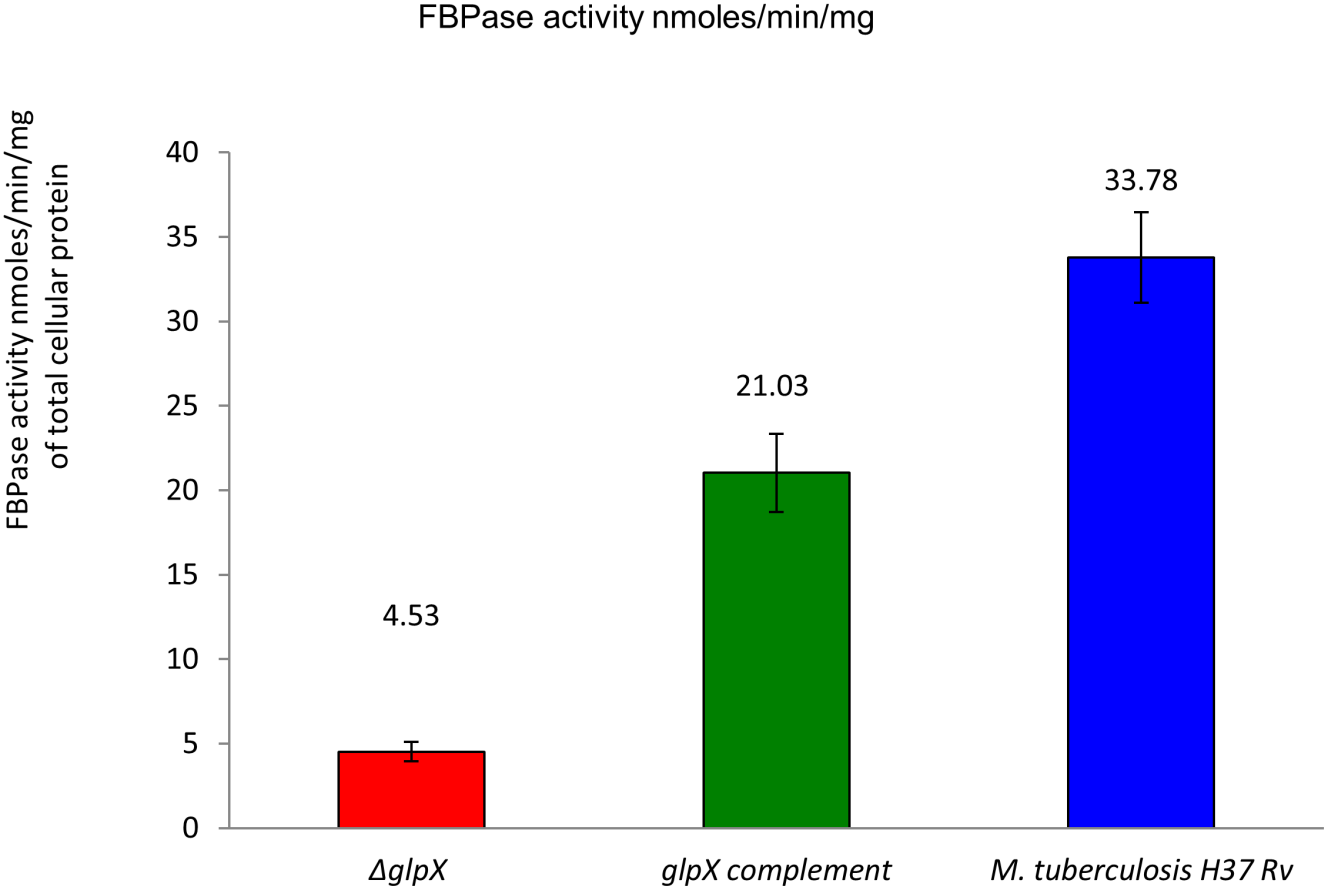
*glpX* gene deletion does not completely abolish the FBPase activity in *Δglpx*: FBPase Activity was measured in nmol/min/mg protein in crude extracts, mean of two determinations, limit of detection = 0.4. All values are with a substrate-free control (no FBP) subtracted. The reported readings are the average of 6 measurements coming from duplicate protein samples (n = 3X2 = 6).

To determine the role of *glpX* encoded FBPase in a model of pulmonary tuberculosis, immune-competent BALB/c mice were infected with aerosolized WT *Mtb*, Δ*glpX*, and the complemented strains. The aerosolization parameters were set and validated such that they ensured an initial pulmonary bacterial load of about 25–75 CFU/mouse. Such an initial instillation of CFU was achieved for the WT *Mtb* and the complemented strain but not for the Δ*glpX* strain, indicating a possible attenuation with regard to infectivity. This difference in initial instillation bacterial count was observed irrespective of the fact that the culture titers of the strains used for infection were all approximately 1 X 10^6^ CFU/ml (data not shown). It is clear that the Δ*glpX* strain failed to efficiently replicate during the acute phase (i.e. first 30 days post-infection) of infection as compared to WT *Mtb*. The CFU for the Δ*glpX* strain were consistently 2–3 log_10_ lower than those for WT *Mtb*. The CFU for Δ*glpX* in lungs started to decline rapidly after day 57 (Fig 6) and continued to remain low 120 and 180 days post-infection. Introduction of *glpX* expressed from its native promoter (*glpX* complement strain) restored survival and replication through both acute and chronic phases, although the bacterial load in the lungs did not reach the WT level (data not shown). Similar to the in vitro growth profile of the *glpX* complemented strain on fatty acids or gluconeogenic growth substrates, growth in vivo was not fully restored to WT levels (Fig 6) Such a behavior has been observed for complemented strains of several mutants [9, 23, 24]. It is noteworthy that a *glpX* gene deletion does not completely abolish FBPase activity in *Mtb* (Fig 7). These experiments demonstrate that *glpX* is not only essential for *Mtb* to establish an infection and to grow during the acute phase of infection, but is equally important for survival of *Mtb* during the chronic phase of infection.

### Fructose 1,6-bisphosphatase activity in cell extracts

The total cellular FBPase activity in WT, ΔglpX and complemented strains indicated that the deletion of *glpX* gene does not completely abolish FBPase activity (*ΔglpX* activity = 4.53 ± 0.57 nmol/min/mg), and while the *glpX* complement strain restored the FBPase activity levels (FBPase activity = 21.03 ± 2.31 nmol/min/mg), it does not restore it to the WT levels (FBPase activity = 33.78 ± 2.67 nmol/min/mg). 2 mM of F1,6BP was used as substrate in this assay.

### *ΔglpX* sensitivity to stress factors

The survival of the knockout under various in vitro stress conditions (low pH, hydrogen peroxide and nitric oxide) was measured and no significant changes were observed in comparison with Wild type (data not shown).

### MIC for *ΔglpX* strain is comparable to that for WT *Mtb* for all the standard drugs tested except for PA-824

The inhibitory concentrations for both strains were not strikingly different except for PA-824 (Table 3).

**Table 3:** MICs for standard antibacterial drugs as determined by the MABA assay.

	MIC (μM)^a^	
Standard drugs	*ΔglpX*	WT *Mtb* H37Rv
RMP	0.023 ± 0.005	0.053 ± 0.007
INH	0.479 ± 0.010	0.476 ± 0.009
MET	> 512	> 512
CAP	1.72 ± 0.061	1.89 ± 0.095
SM	0.48 ± 0.097	0.82 ± 0.083
**PA-824**	**0.48 ± 0.027**	**0.16 ± 0.007**
OPC-67683	0.006 ± 0.001	0.009 ± 0.001

^a^ Reported MIC values are an average (± standard deviation) of 6 independent assays. Although the MIC of PA-824 for the *ΔglpX* strain is significantly higher (about 3–4 fold) than that observed for WT *Mtb* strain, it is within the normal MIC range of 0.015 to 0.5 μM.

## Discussion

As expected, in aerated batch cultures, WT *Mtb* strain grew fastest and achieved the highest growth rate on glycerol, followed by dextrose and acetate, similar to the earlier studies [25]. While the ability of *ΔglpX* to grow on glycerol or acetate as a sole carbon source was severely compromised as compared to WT *Mtb*, *ΔglpX* grew twice as fast as WT *Mtb* on dextrose as a sole carbon source. The slower growth profile of *ΔglpX* on glycerol or acetate was expected since *glpX* encodes a functional fructose 1,6-bisphosphatase (FBPase) required for growth on gluconeogenic substrates (glycerol/fatty acids/acetate) [10, 20]. Previous studies [4, 26–30] indicate that *Mtb* grows fastest in vitro on glycerol, less quickly on dextrose, and least quickly on acetate.

The fast growth of *ΔglpX* on dextrose is most likely a result of the disruption of a possible regulatory mechanism of FBPase with the phosphofructokinase (encoded by *pfk1* and *pfk2* in *Mtb*) enzyme (which catalyzes the reverse reaction converting fructose 6-phosphate to fructose 1,6-bisphosphate). However the presence of such a regulatory mechanism for FBPase is not experimentally verified in *Mtb*. Taken together the observations indicate the *ΔglpX* does not display an eugonic growth phenotype on gluconeogenic substrates (acetate, glycerol, fatty acids), rather it is dysgonic. This observation is concordant with the expected disruption of FBPase activity in *Mtb* by deletion of the *glpX* gene. The growth of WT *Mtb* on an equimolar mixture of glycerol and acetate exceeded that achieved with either constituent alone. Similar effects were observed using a mixture of dextrose and glycerol and a mixture of acetate and dextrose. The compromised growth of *ΔglpX* on glycerol or acetate was not completely rescued by their combination either. However, with the presence of dextrose in the medium (dextrose + glycerol or dextrose + acetate), the growth profile was almost similar to WT *Mtb*. A reason for such a phenotype is that both glycerol and acetate are gluconeogenic in nature and therefore cannot be effectively utilized by the *ΔglpX*. However, dextrose being a preferred glycolytic nutrient for *ΔglpX* easily rescues the *glpX* mutant strain and the combinations involving dextrose grew in a manner similar to WT *Mtb*. Central carbon metabolism in *Mtb* is a peculiar case involving compartmentalization of metabolic pathways and thereby the metabolites. As understood from our results and the model of compartmentalized central carbon metabolism [25], *Mtb* preferentially utilizes glycerol over dextrose and acetate. The predominant pattern of distribution for glycerol and acetate combination as carbon source requires a functional *glpX/*FBPase. However, since *ΔglpX* does not possess a fully functional FBPase, its growth is severely compromised on gluconeogenic substrates like glycerol and acetate. The reduced growth/growth-compromised phenotype of *ΔglpX* is rescued to some extent by dextrose in combination growth media since with dextrose as a carbon source, FBPase activity becomes dispensable (predominant pattern of distribution is not gluconeogenic but rather glycolytic).

As anticipated, *glpX* gene deletion adversely affected the FBPase activity levels in Δ*glpX*. However, FBPase activity was not completely abolished in the Δ*glpX* indicating a possible compensation by other genes/alternate proteins having FBPase activity. The residual FBPase activity in *ΔglpX* can be attributed to several other gene products/ other classes of enzymes such as inositol monophosphatases (IMPase). The *M*. *tuberculosis* genome encodes four IMPase like genes, ImpA, SuhB (*Rv2701c*), ImpC (*Rv3137*) and CysQ (*Rv2131c*) [15, 31, 32]. The SuhB of *Mtb* has been extensively characterized both structurally and biochemically [31, 33]. SuhB appears to be a bona fide IMPase with no activity towards fructose-1,6-bisphosphate. The purified CysQ *(Rv2131c)* gene product, in addition to having IMPase and FBPase activity, showed substrate specificity that was broader than those of several bacterial and eukaryotic IMPase. The dimeric enzyme exhibited dual activities of IMPase and FBPase, with K_m_ of 0.22 ± 0.03 mM for IMP and K_m_ of 0.45 ± 0.05 mM for FBP. All four IMPases in *Mtb* share very limited sequence homology with each other (<30% identity with the best being SuhB which is 30% identical to ImpC (*Rv3137*)). Both ImpA and ImpC share similar sequence homology (20–25% sequence identity) with TM1415 IMPase/FBPase–*Thermotoga maritime* (PDB id: 2P3N) [34] and MJ0109 IMPase/FBPase–*Methanococcus jannaschii* (PDB id: 1G0H) [35]. It is also noteworthy that Both ImpA and ImpC have not been characterized structurally or biochemically yet to rule out the possibility of a dual function IMPase/FBPase. Taken together the residual FBPase activity in *ΔglpX* is attributable to CysQ or ImpA and ImpC.

The in vivo survival profile suggests that *glpX* is required to achieve an initial instillation dose and also maintain high bacterial loads at later time points (acute and chronic phases). The initial instillation dose for *ΔglpX* is significantly lower than the WT *Mtb* and complement respectively. There are several reports wherein the initial bacterial loads of certain gene deletion strains of *Mtb* are significantly different than the control WT *Mtb* strain [24, 36, 37]. In the study by Venugopal *et al*., in describing the phenotype of *ΔdlaTΔpdhC*, the deletion strain was not recovered post-day one despite equal input into the aerosolizer. However, by day 7, the *ΔdlaTΔpdhC* and *ΔlpdC* mutants were both recovered at 100 CFU, suggesting that the *ΔdlaTΔpdhC* mutant was viable but non-culturable at day one. We expect a similar case for Δ*glpX*, where the deletion of *glpX* has limited the viability of the bacteria. The lower bacterial counts at later time points could be due to an initially low instillation dose (<100 CFU at 1 day post infection), however the rate of growth differs during the initial 30 days post-infection. The significance or essentiality of genes can be efficiently studied at varying stages of disease progression (acute vs. chronic infection) by generating conditional mutants to understand the function of such essential genes. Δ*glpX* strain like WT *Mtb*, is sensitive to stress factors likely to be encountered inside the host, such as low pH, hydrogen peroxide, and nitric oxide indicating that the growth suppressed phenotype in vivo is primarily due to a drastic reduction of FBPase activity and not to any other underlying effects.

The Δ*glpX* strain showed similar MICs for all the drugs tested except for PA-824. The *glpX* gene deletion reduces the inhibitory activity of PA-824 but not OPC-67683, a structurally similar nitroimidazole. *Rv3547*, encoding a 151 amino acid protein with no similarity to any protein with a known function, was characterized as a F420-dependent nitroreductase [38, 39]. F420-dependent glucose-6-phosphate dehydrogenase, which catalyzes the oxidation of G6P to 6-phosphogluconolactone, is required for the intracellular reduction of the deazaflavin cofactor F420, which serves as the hydride donor to PA-824 in the *Rv3547* catalyzed reduction of this compound. Mutations in the mycobacterial *Rv3547* gene are found in OPC-67683-resistant *Mtb* strains as well, suggesting that the *Rv3547*-encoded enzyme is required for activation of both PA-824 and OPC-67683 [40]. However, there is no conclusive evidence whether FGD1 and coenzyme F420 are also needed for activation of OPC-67683, since there were no OPC-67683 resistant strains with variations (mutations) in FGD1 or coenzyme F420. Therefore it is possible that the activation mechanism of OPC-67683 is FGD1/F420 independent which would be consistent with our results wherein the MIC of OPC-67683 for *ΔglpX* strain is comparable to that of WT *Mtb* strain. The reduced FBPase activity in *ΔglpX* strains could result in lower intracellular F6P/G6P levels and hence reduced G6P dehydrogenase activity and the corresponding reduced hydride donor activity of F420. Thus the modest resistance of *ΔglpX* to PA-824 may be due to reduced intracellular G6P/F6P levels.

In conclusion the *glpX* gene encodes a functional FBPase and is essential for both in vitro and in vivo growth and survival of *Mtb*. The loss of this gene results in drastic reduction of FBPase activity but not complete abolition. Our findings verify the *glpX* encoded FBPase II in *Mtb* can be a potential target for drug discovery.
